# Meta-analysis of economic evaluation studies: data harmonisation and methodological issues

**DOI:** 10.1186/s12913-022-07595-1

**Published:** 2022-02-15

**Authors:** Bhavani Shankara Bagepally, Usa Chaikledkaew, Nathorn Chaiyakunapruk, John Attia, Ammarin Thakkinstian

**Affiliations:** 1 Mahidol University Health Technology Assessment (MUHTA) Graduate Program, Mahidol University, Bangkok, Thailand; 2 ICMR-National Institute of Epidemiology, Chennai, India; 3 Social Administrative Pharmacy Division, Department of Pharmacy, Faculty of Pharmacy, Mahidol University, Bangkok, Thailand; 4 Department of Pharmacotherapy, College of Pharmacy, University of Utah, Utah, USA; 5 Centre for Clinical Epidemiology & Biostatistics, School of Medicine and Public Health, Hunter Medical Research Institute, University of Newcastle, New Lambton, NSW Australia; 6 Department of Clinical Epidemiology and Biostatistics, Faculty of Medicine Ramathibodi Hospital, Mahidol University, Rama VI Road, Bangkok, 10400 Thailand

**Keywords:** Economic evaluation, CUA, Cost-effectiveness

## Abstract

**Background:**

In the context of ever-growing health expenditure and limited resources, economic evaluations aid in making evidence-informed policy decisions. Cost-utility analysis (CUA) is often used, and CUA data synthesis is also desirable, but methodological issues are challenged. Hence, we aim to provide a step-by-step process to prepare the CUA data for meta-analysis.

**Methods:**

Data harmonisation methods were constructed specifically considering CUA methodology, including inconsistent reports, economic parameters, heterogeneity (i.e., country’s income, time horizon, perspective, modelling approaches, currency, willingness to pay). An incremental net benefit (INB) and its variance were estimated and pooled across studies using a basic meta-analysis by COMER.

**Results:**

Five scenarios show how to obtain INB and variance with various reported data: Study reports the mean and variance (Scenario 1) or 95% confidence interval (Scenario 2) of ΔC, ΔE, and ICER for INB/variance calculations. Scenario 3: ΔC, ΔE, and variances are available, but not for the ICER; a Monte Carlo was used to simulate ΔC and ΔE data, variance and covariance can be then estimated leading INB calculation. Scenario-4: Only the CE plane was available, ΔC and ΔE data can be extracted; means of ΔC, ΔE, and variance/covariance can be estimated accordingly, leading to INB/variance estimates. Scenario-5: Only mean cost/outcomes and ICER are available but not for variance and the CE-plane. A variance INB can be borrowed from other studies which are similar characteristics, including country income, ICERs, intervention-comparator, time period, country region, and model type and inputs (i.e., discounting, time horizon).

**Conclusion:**

Out data harmonisation and meta-analytic methods should be useful for researchers for the synthesis of economic evidence to aid policymakers in decision making.

**Supplementary Information:**

The online version contains supplementary material available at 10.1186/s12913-022-07595-1.

## Background

In the context of ever-growing health expenditure and limited resources, identifying healthcare services that yield the highest benefit at the lowest cost is a priority. Economic evaluation studies (EES) provide a framework to systematize both clinical and economic outcomes [1] Cost-utility analysis (CUA) is commonly applied to compare clinical and economic outcomes by estimating an incremental cost-effectiveness ratio (ICER). The costs are usually measured in a specific country currency, while the health benefit is usually measured as a quality adjusted life year (QALY), i.e., the product of years lived and health utility score ranging from 0 (death) to 1 (perfect health), or disability adjusted life years (DALY) [2, 3]. The ICER, (Cost_intervention_—Cost_comparator_)/(QALY_intervention_—QALY_comparator_), is under the willingness to pay (WTP) threshold (measured in monetary cost per QALY gained), the health intervention is considered to be cost-effective[4]. The guidelines from Joanna Briggs Institute, Cochrane group [5, 6], mainly provide guidelines towards qualitative synthesis or only systematic review of all sorts of economic evaluation (e.g., cost–benefit analysis, cost minimisation analysis, cost-effective analysis, cost-utility analysis) [7–11]. However, these guidelines have a limited focus on data extraction and data harmonisation process to prepare the data for the meta-analysis [7–11].

Further, many methodological issues in the data synthesis of EESs are more challenging than clinical studies because there are many sources of heterogeneity, including study characteristics (e.g., setting, WTP, country, country income), methodology (time horizon, perspective, data source, model type, input parameters, and assumptions) [8]. This is perhaps why most previous systematic reviews of EESs have performed only descriptive analyses and reported only qualitative findings without applying a meta-analysis (MA) to estimate pooled effect measures.

Although Crespo et al. [8] have described a MA for pooling EES (known as the COMparative Efficiency Research, COMER), it has yet been widely adopted as such MA for clinical outcomes. This might be due to EESs being too heterogeneous to pool or choosing the lesser-known parameter “incremental net benefit” (INB) as the effect measure rather than the more commonly used ICER. However, we believe the choice for pooling INB was justified due to the limitations of the ICER [12]. For instance, a negative ICER may indicate a lower cost compared with higher effectiveness or higher costs along with lower effectiveness of interventions, thus introducing ambiguity in interpretation [8, 13]. In contrast, positive and negative INBs directly indicate cost-effectiveness and non-cost-effectiveness of interventions, respectively, which is the information required by policymakers [14, 15].

Furthermore, the COMER method mainly focused on the statistical methods for pooling but did not suggest a detailed step-by-step method of data extraction and data harmonisation as for various styles and suboptimal quality for reporting of economic evaluations [16]. In addition, assessing heterogeneity and exploring the source of it and publication bias have not been described. Therefore, our primary focus in this manuscript is to provide a methodological approach for meta-analysis of cost-utility studies; we have specifically detailed the step-by-step process to extract data from “Cost-utility studies” and to make it ready to be taken for meta-analysis. Data for the cost-effectiveness of diabetic drug controls are used as a demonstration.

## Methods

Basic methods of MA in EESs, including identifying and selecting relevant studies, are similar to other systematic reviews and MAs [5, 17] and should follow the Preferred Reporting Items for Systematic Reviews and Meta-Analyses (PRISMA) [18] guideline when reported. This methodological study was a part of previous MAs, in which some additional specific issues apply to EESs are as follows [19–22]; the relevant protocol was registered in Prospero (PROSPERO 2018 CRD42018105193).

### Step 1: Data extraction

All relevant data for comparative EESs (e.g., CUA) should be extracted as follows utilising the Population, Intervention, Comparator, Outcome, and Study type (PICOS) framework:

- General characteristics of EESs including study setting/country, study design (e.g., CUA with model-based, primary CUA alongside RCT/cohort), study perspective, time horizon, discount rate for cost and utility, currency/currency-year, type of EESs [e.g., CUA or cost-effectiveness analysis (CEA)], willingness to pay [WTP; country-specific or gross domestic products (GDP)-base] or country-level cost-effectiveness threshold [23] where appropriate if WTP was not available, and type of economic models.
- Characteristics of patients (P) including indication for treatment, sample size, type of patients (e.g., children/adult, general/specific disease, etc.), mean age, percent male/female, mean body mass index (BMI), etc.
- Type of interventions and comparators (I & C) (with the duration of treatment/dosage/day, etc.)
- Data needed to estimate INB and its variance (O); this includes currency and year, source of cost data (actual data cost collected from patients, central hospital/country costs, etc.), type of cost (e.g., direct/non-direct medical cost, indirect medical cost), and effectiveness outcomes (e.g., life year, QALY, improvement, success, adverse events etc.).

Specific data required for pooling include costs or incremental cost (ΔC), and incremental effectiveness (ΔE) along with their standard deviation (SD), standard error (SE), or 95% confidence interval (CI) along with covariance between ΔC and ΔE. Some studies may report ICER along with probabilistic sensitivity analysis (PSA). To calculate the INB and its variance, mean and variance of the costs and effectiveness of interventions and comparators along with WTP thresholds are required. In the model-based CUA, studies usually report point estimates of deterministic and/or probabilistic costs and outcomes. We suggest using primarily the measures of central tendency and dispersion measures from PSA results for pooling, as it could better represent a real-life situation considering the distribution of all input variables. Further, to conduct sensitivity analyses using point estimates from the deterministic analysis to see the robustness of results. The WTP threshold was initiated by the Commission on Macroeconomics and Health in 2002 by the World Health Organization CHOosing Interventions that are Cost-Effective (WHO CHOICE) [24]. The WTP threshold in each country usually refers to the standard country guideline based on a fixed value or per capita GDP with returns on investments in health to define whether a health intervention would be (very) cost-effective. [25, 26]. We suggest using the same WTP threshold in monetary units used in the study with further adjustment as per currency conversions as mentioned below. If studies have not reported WTP, one per capita GDP of that study’s country and year can be used as WTP along with a sensitivity analysis of three times per capita GDP.

We strongly recommend constructing data extraction forms in advance, a pilot should be performed to make sure that the forms work well and contain all important data specific to that topic.

### Step 2: Data harmonisation

#### Currency conversions

We need to standardize money units usually reported in different currencies (i.e., US $, €, £, ¥) and years by converting to purchasing power parity (PPP) adjusted to US$ for the latest year of analysis[8]. For example, if a study reported cost, ICER, and thresholds in Euros for 2012 and we plan to pool for the current year (e.g., 2022), this currency is firstly converted to 2022 Euros using the historical consumer price index (CPI) of that country (IMF database: https://www.imf.org/en/Publications/WEO/weo-database/2021/October/download-entire-database). Then, the Euro 2022 value to be converted to PPP adjusted US$ rate using conversion rates from the International Monetary Fund [27]. In addition, GDP-based WTP threshold (K) values also need to be corrected for the current CPI 2022 year and PPP; however, standard/country-specific or fixed WTP values only need PPP correction. The variance is calculated as follows:

1 $$Va{r}_{{Adjusted}_{2022}}=Va{r}_{Euro{s}_{2012}}x{\left(\frac{CP{I}_{Euro{s}_{2022}}}{CP{I}_{Euro{s}_{2012}}}x\frac{1}{PP{P}_{2022}}\right)}^{2}$$

#### Estimation of INB and its variance

After currency conversions for cost and K, the INB can be further estimated as follows [8]:

2 $$INB=Kx\Delta E-\Delta C$$

or

3 $$INB=\Delta E(K-ICER)$$

Where K is the WTP, and ΔC and ΔE are incremental cost and incremental effectiveness, respectively.

A positive INB favours treatment, i.e., intervention is cost-effective, whereas a negative INB favours the comparator, i.e., intervention is not cost-effective [8, 14, 15].

The variance of INB [8] can be estimated as follows:

4 $$Var\left(INB\right)={K}^{2}{\sigma }_{\Delta E}^{2}+{\sigma }_{\Delta C}^{2}-2K{\sigma }_{\Delta E\Delta C}$$

or

5 $$Var(INB)={K}^{2}{\sigma }_{\Delta E}^{2}+{\sigma }_{ICER}^{2}$$

**Fig. 1:**
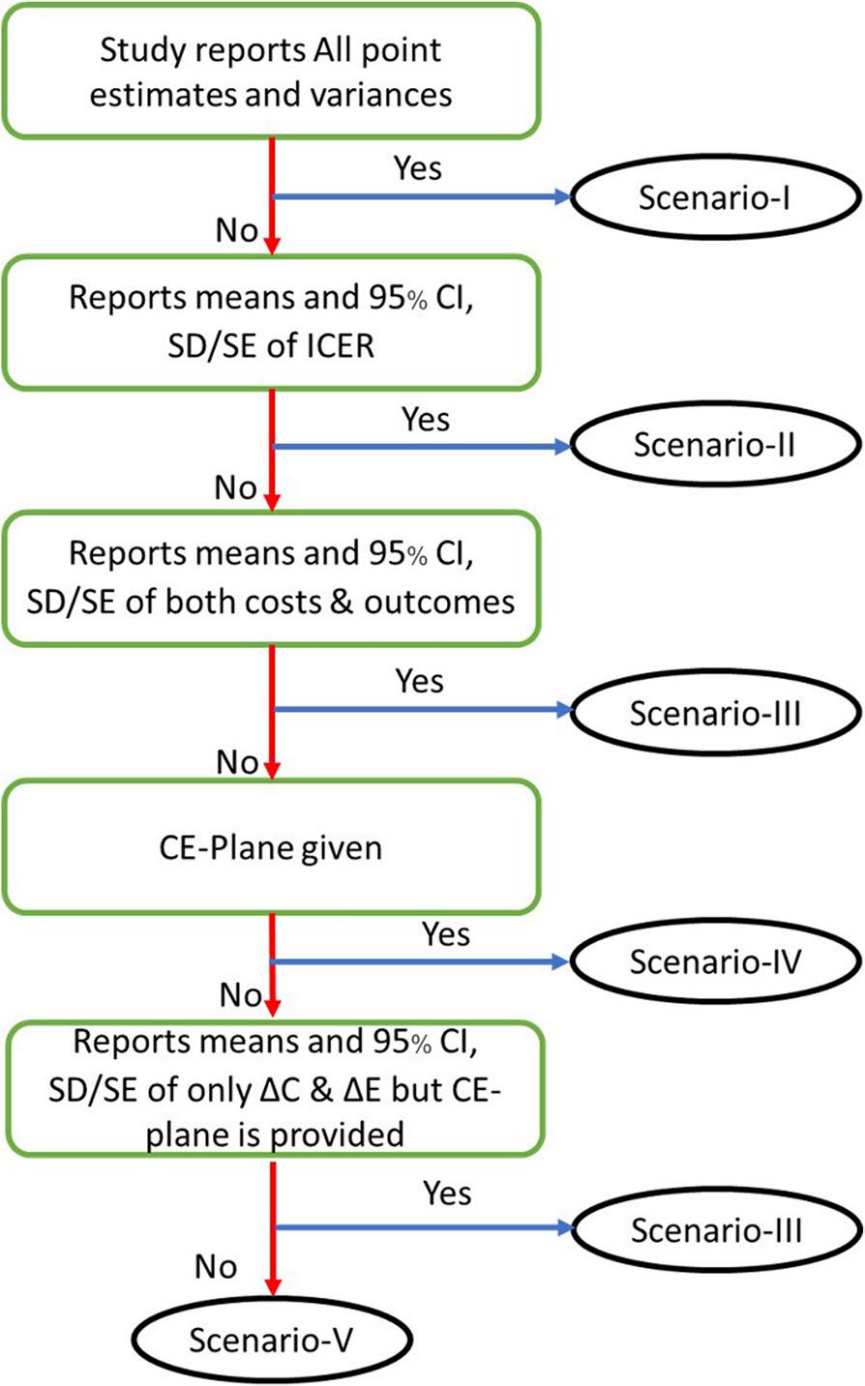
Selection flow chart of scenarios

Where ${\sigma }_{\Delta C}^{2}, {\sigma }_{\Delta E}^{2}, {\upsigma }_{\Delta E\Delta C}$ are variances of ΔC and ΔE and their covariance, and ${\sigma }_{ICER}^{2}$ is variance of ICER. However, economic studies can report many different parameters; the five scenarios below, along with a flow in Fig. 1, shows how to obtain INB and variance starting with different reported data [28].

##### Scenario-1

The primary EES ideally reports the point estimates and variances for every parameter required for the calculation of INB and its variance. The INB can be calculated accordingly to Eqs. (2) to (5).

##### Scenario-2

The study reports the means and measures of dispersion (95% CIs) of incremental costs & outcomes and ICER. The variance of the ICER can be calculated using the following formulas:

6 $$\begin{array}{c}95\mathrm{\%}CI of {\mu }_{ICER}={\widehat{\mu }}_{ICER}\pm {Z}_{a/2}xSE\\ {UL}_{ICER}={\widehat{\mu }}_{ICER}+{Z}_{a/2}xSE\\ \begin{array}{c}SE=\frac{{UL}_{ICER}-{\widehat{\mu }}_{ICER}}{{Z}_{a/2}}\\ \begin{array}{c}{\widehat{\sigma }}_{ICER}^{2}={SE}^{2}\\ {UL}_{ICER}=\mathrm{Upper limit of ICER}\\ \begin{array}{c}{Z}_{a/2}=\mathrm{Standardize normal}=1.96\mathrm{ if alpha}=0.05\\ {\widehat{\mu }}_{ICER}=\mathrm{mean ICER}\end{array}\end{array}\end{array}\end{array}$$

Once we know the variance of the ICER, the variance of the INB can be estimated using Eq. (5).

##### Scenario-3

The study reports means, and 95% CI, SD/SE of costs/outcomes, or ΔC/ΔE, but does not provide the ICER or its variance. Data for Costs/ΔC and QALYs/ΔE are then used to simulate Costs/ΔC and QALYs/ΔE with 1000 replications using Monte Carlo methods with gamma and normal distributions for Costs/ΔC and QALYs/ΔE, respectively. We suggest simulating costs and QALYs of intervention and comparator for 1000 replications separately; this then leads to calculating the ΔC, ΔE, and covariance between ΔC & ΔE. Data for ΔC and ΔE are assumed to be gamma and normal distributions[29, 30].A sensitivity analysis can be performed using different distributions (e.g., log-normal, exponential for both costs and effective, etc.) to see the robustness of pooling results. The covariance (${\upsigma }_{\Delta E\Delta C}$) between ΔC and ΔE as well as ${\widehat{\sigma }}_{\Delta C}^{2}$ & ${\widehat{\sigma }}_{\Delta E}^{2}$ can be then estimated. If the 95% CI is provided, this is converted to SE using Eq. (6) above and used to simulate data. The INB and its variance can be further calculated using Eqs. (2) and (5).

##### Scenario-4

The study does not report any dispersion but does provide the CE plane graphs, a scatter plot of ΔC on the Y-axis and ΔE on the X-axis, in which individual values of ΔC and ΔE data can be manually extracted from the CE plane using Web-Plot-Digitizer software[31]. Then, means of ΔC, ΔE, and their variances and covariances can be estimated accordingly. Finally, the INB and its variance can be estimated using Eqs. (2) and (5).

##### Scenario-5

The study reports neither any dispersion nor the CE-plane graph but only provides the deterministic analysis means (or point estimates) of costs, outcomes, and ICER. In such situations, the measures of dispersions can be borrowed from another similar study if they fulfill the following criteria:

- They are in the same stratum of country income,
- Their ICERs are not much different, e.g., ± 50% to 75%
- They are similar in intervention, comparator, time period, country region
- Similar model type and inputs (i.e., discounting, time horizon).

If there is more than one study that meets the criteria, the average of the variances of those studies can be used.

### Step 3: Pooling INB

When pooling INBs from many studies, we strongly recommend stratifying by the level of country income, model type, time horizon, and perspective in order to reduce heterogeneity. The country income should be classified as low (LIC), lower-middle (LMIC), upper-middle (UMIC), and high (HIC) as per the World Bank classification^8^. Economic models can include Markov, decision tree, discrete event simulation, or others. Study perspectives should include societal, third-party payer, and patient perspectives. Time horizon should be lifetime (e.g., ≥ 20 or 30 years depending on the disease context) and non-lifetime (e.g., < 5, < 10-years, etc.).

The INB can be pooled across studies using a fixed-effect or a random-effect model depending on the degree of heterogeneity [5, 8, 19, 20, 28].

A) Fixed-effects model
  7 $${\mathrm{INB}}_{\mathrm{p}}=\frac{\sum_{\mathrm{i}=1}^{\mathrm{S}}{\mathrm{w}}_{\mathrm{i}}.{\mathrm{INB}}_{\mathrm{i}}}{\sum_{\mathrm{i}=1}^{\mathrm{S}}{\mathrm{w}}_{\mathrm{i}}}$$
  8 $${w}_{i}=\frac{1}{Var\left({INB}_{i}\right)}$$
B) Random-effects model
  9 $${\mathrm{INB}}_{\mathrm{p}}=\frac{\sum_{\mathrm{i}=1}^{\mathrm{S}}{w}_{i}^{*}.{\mathrm{INB}}_{\mathrm{i}}}{\sum_{\mathrm{i}=1}^{\mathrm{S}}{w}_{i}^{*}}$$
  10 $${w}_{i}^{*}=\frac{1}{Var({INB}_{i})+{\tau }^{2}}$$
  11 $${\tau }^{2}=\frac{Q-(S-1)}{\sum {w}_{i}-\frac{\sum {w}_{i}^{2}}{\sum {w}_{i}}}$$

where Q is the Cochrane Q test, which has Chi-square distribution; S is a number of included studies/comparisons; τ^2^ is a between-study variation.

Similar to MA in other areas, heterogeneity needs to be assessed before pooling INB. Heterogeneity can be visualized by inspection of the forest plot and quantitated using the Cochrane-Q test and the I^2^ statistic[5].

12 $$\mathrm{Q}=\sum_{\mathrm{i}=1}^{\mathrm{S}}{\mathrm{w}}_{\mathrm{i}}{\left({\mathrm{INB}}_{\mathrm{i}}-{\mathrm{INB}}_{p}\right)}^{2}$$

13 $${\mathrm{I}}^{2}=100\mathrm{\%x }\frac{\mathrm{Q}-(\mathrm{S}-1)}{\mathrm{Q}}$$

If heterogeneity is present, i.e., the I^2^ ≥ 25% or *p*-value of Q test is less than 0.1; the INBs can be pooled using a random-effects model; otherwise, a fixed-effect model can be applied [8, 19, 20, 28]. Exploring source/s of heterogeneity is strongly recommended. This can be done using a meta-regression to fit each potential source (e.g., time horizon, percent discount rate, threshold values, source of effectiveness measure, risk of bias, economic structure, etc.) one-by-one [8, 19, 20, 28]. If that potential factor explains some proportion of the heterogeneity, including it in the meta-regression model should reduce the I^2^ accordingly. There are no established criteria for how much I^2^ should be decreased to consider that factor as a significant source of heterogeneity. In our experience, if the I^2^ is reduced by about 50% or more from the baseline model (i.e., the model without any factor), such factor/s may be source/s of heterogeneity. A post-hoc subgroup analysis by that factor should be performed accordingly. In addition, sensitivity analyses excluding a few studies with very different characteristics compared to the rest can be used to see if heterogeneity of INBs can be reduced.

Similar to general MA, publication bias should be assessed using a funnel plot and Egger's test. A funnel plot graphs INB estimates on the x-axis against their precision on the y-axis. If all studies are estimating the same true INB, their INBs should be randomly scattered around the true value and form a funnel shape. Egger’s test formally tests if the funnel is symmetrical; if this is significant, it usually indicates that there is heterogeneity or missing studies (publication bias) or both. A contour-enhanced funnel plot is further recommended [32]. This plot will contour the area of the funnel into non-significant (*P*-value > 0.05- < 0.1) and significant areas (*P*-value < 0.01 and < 0.05), which will help to differentiate the cause of the asymmetry. For instance, if missing studies fall into the non-significant area, asymmetry might be due to missing studies or publication bias. Conversely, if missing studies are in significant areas, heterogeneity is more likely to be the explanation.

### Example

We used data from a MA of CUA of glucagon-like peptide 1 agonists (GLP1) for treatment of type 2 diabetic (T2D) patients who failed to achieve control with metformin monotherapy [19]. A total of 56 studies with 82 comparisons were eligible for pooling INBs. We included comparisons of GLP-1 and dipeptidyl peptidase-4 inhibitors (DPP4i) (*N* = 10); study characteristics are described in Table 1. All studies were from HICs and used country-specific WTP threshold; 9/10 studies used the third-party payer perspective, and 7/10 used a lifetime horizon.

**Table 1:** Selected studies and its analysis scenario

**Study**	**Country**	**Perspective**	**Time horizon**	**Reference year**	**Threshold**	**Coversion factor**	**ICER**	**Measure of dispersion**	**CE-Plane**	**Scenario**
Sinha [33]	USA	Payers	Life-time	2008	$ 50,000	1.138489257	yes	none	no	5
Davies [34]	UK	Payers	Life-time	2008	£ 20,000	1.745246374	yes	SD	no	3
Guillermin [35]	USA	Payers	35-yrs	2010	$ 50,000	1.124115573	no	SD	no	5
Lee [36]	USA	Payers	35-yrs	2011	$ 50,000	1.089714997	yes	SD	no	3
Mezquita-Raya [37]	Spain	Payers	Life-time	2012	€ 30,000	1.562673067	yes	SD	yes	3
Steen-Carlsson [38]	Sweden	Societal	Life-time	2013	SEK 500,000	0.116048611	yes	NA	yes	4
Perez [39]	Spain	Payers	Life-time	2012	€ 30,000	1.562673067	yes	SD	no	3
Bruhn [40]	USA	Payers	50-yrs	2014	$ 50,000	1.035412488	yes	SD	yes	3
Roussel [41]	France	Payers	Life-time	2013	€ 30,000	1.261085687	yes	95% CI & SD	yes	3
Barnett [42]	UK	Payers	Life-time	2016	£ 20,000	1.46911077	yes	SD	yes	3

*CI* confidence interval, *SD* Standard deviation

In terms of preparing the data for pooling, 7, 1, and 2 studies provided data matching scenarios 3, 4, and 5, respectively (Table 1). Data for mean cost, QALY, and their incremental values are described in Table 2. Costs and WTP thresholds from each study were converted to $US currency using PPP adjusted for the year 2019 using formula (1).

**Table 2:** Descriptive of the mean cost and QALY along with their incremental data of comparison between GLP1a vs DPP4i

Author	Cost				QALY			ICER
	**Currency**	**GLP1**	**DPP4i**	**ΔC**	**GLP1**	**DPP4i**	**ΔE**	
Sinha [33]	US $	170,799	167,163	3636	15.2998	15.3335	-0.0337	-107,893
Davies [34]	£	21,793 ± 544	19,951 ± 521	1842 ± 751	7.52 ± 0.11	7.34 ± 0.11	0.19 ± 0.15	10,158
Guillermin [35]	US $	55,647	57,862	-2215	9.56 ± 0.12	9.28 ± 0.12	0.284 ± 0.172	-7799
Lee [36]	US $	81,444 ± 1079	76,262 ± 1061	5182	8.825 ± 0.117	8.624 ± 0.115	0.201	31,488
Mezquita-Raya[37]	€	54,684 ± 1250	52,387 ± 1346	2297	9.04 ± 0.13	8.87 ± 0.11	0.17	13,266
Steen-Carlsson [38]	SEK	1,360,715	1,304,092	56,624	10.53	10.15	0.38	154,226
Perez [39]	€	56,628 ± 1323	52,450 ± 1394	4177	9.239 ± 0.121	8.838 ± 0.121	0.4	10,436
Bruhn [40]	US $	140,806 ± 1948	138,583 ± 2071	2223	9.618 ± 0.125	9.517 ± 0.130	0.101	22,094
Roussel [41]	€	43,031 ± 1532	40,472 ± 1513	2558 (2427,2689) ^a^	10.09 ± 0.13	9.84 ± 0.13	0.25 (0.24, 0.26)^a^	10,275
Barnett [42]	£	24,737 ± 739	22,362 ± 725	2375	9.18 ± 0.12	9.02 ± 0.11	0.15	15,423

Values in cell are mean ± standard deviation, ^a^95% CI, *ΔC* incremental cost, *ΔE* incremental QALY, *GLP1a* Glucagon-like peptide 1 agonists, *DPP4i* Dipeptidyl peptidase-4 inhibitors

For the seven studies matching scenario 3 (where mean C and E data along with SDs were reported) (Table 1 and Supl Table 1), the Monte-Carlo method was used to simulate 1000 replicated data based on gamma and normal distributions for cost and QALY data, respectively. Then, ΔC and ΔE along with variance and covariance (${\upsigma }_{\Delta E\Delta C})$) were calculated. The INB and variance were then calculated following formulas (2) and (4).

The study matching scenario 4 provided CE-plane graphs (Table 1). Data for ΔC and ΔE were directly extracted from the CE plane using Web-Plot-Digitizer [31]. Then, variance and covariance (${\upsigma }_{\Delta E\Delta C}$) were calculated, leading to estimation of the INB and its variance using formulas (2) and (4).

For the two studies [33, 35] matching scenario 5, the INB variance was adopted from other studies following the steps outlined above. Of the ten included studies, two other studies [36, 40] were conducted in the USA. For the selection of variance values for the Guillermin et al. study[35], the study period, time-horizon, study perspective, ICER values, drug comparison (Sitagliptin) were most similar to Lee et al. [36] (Table 1 and Table 2). Hence, the INB variance value of the latter was used to estimate the former. The values for Lee et al. also matched the second study [33] most closely.

**Fig. 2:**
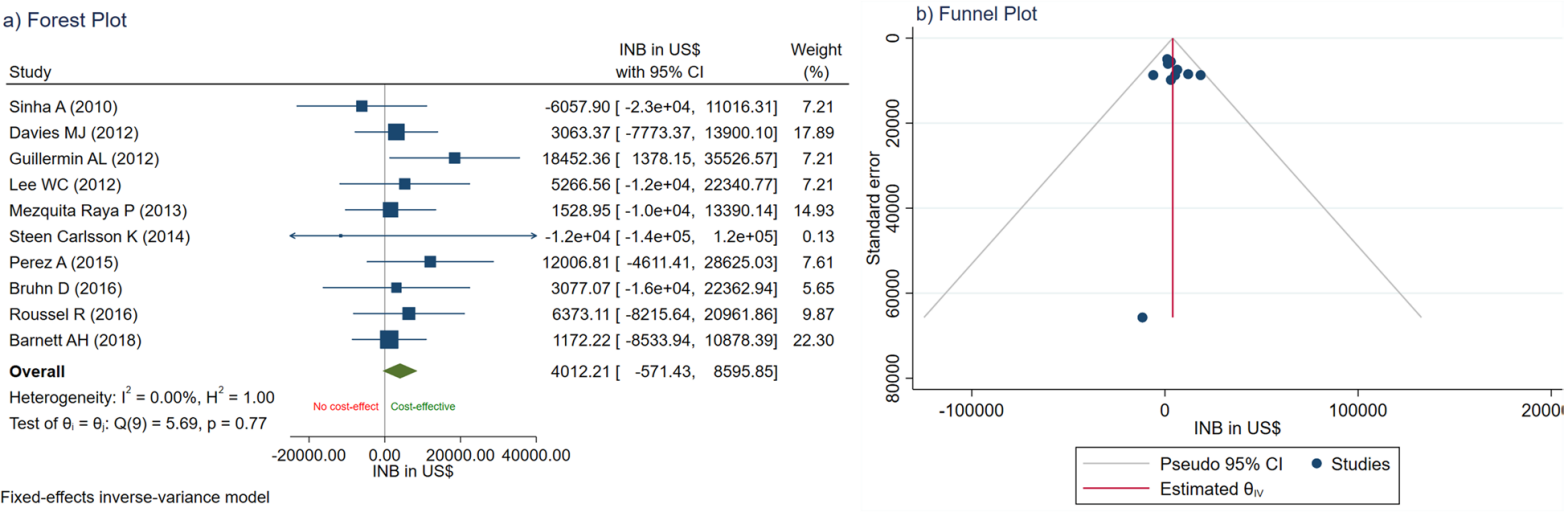
**a** Forest plot of pooling INBs of GLP1 vs. DPP4i; **b** Funnel plot of pooling INB of GLP1 vs. DPP4i

INB data along with variances are shown in Table 3. The forest plot was constructed by plotting point estimated INBs along with 95% CIs for individual studies (see Fig. 2a); the intervention is cost-effective if the estimated INB falls in the right side of a vertical line of zero and it is not cost-effective if it lines in the left side. These INBs were then pooled across studies using a fixed-effects (inverse variance) model yielding a pooled INB (95% CI) of US$ 4012.21 (-571.43, 8595.85) with I^2^ of 0% (see Fig. 2a), this corresponded to individual 95% CIs of INBs which are very much overlapped indicating less likely to be heterogeneous. In the presence of heterogeneity, as indicated by I^2^ ≥ 25% or Cochrane-Q *p* < 0.1, a random-effects model (DerSimonian and Laird model) could be used [43]. The pooled INB value is positive but its 95% CI covers 0, i.e., GLP1 agonists might be cost-effective as compared to DPP4 inhibitors but the results did not reach statistical significance.

**Table 3:** Describe incremental net benefit comparing GLP1i with DPP4i along with variance

Authors	Mean INB (PPP adjusted US $)	Variance INB
Sinha [33]	-6,058	7,58,90,095
Davies [34]	3,063	3,05,70,369
Guillermin [35]	18,452	7,58,90,095
Lee [36]	5,267	7,58,90,095
Mezquita-Raya [37]	1,529	3,66,23,523
Steen-Carlsson [38]	-11,643	4,31,66,49,739
Perez [39]	12,007	7,18,90,710
Bruhn [40]	3,077	9,68,23,864
Roussel [41]	6,373	5,54,03,868
Barnett [42]	1,172	2,45,24,439

*INB* incremental net benefit, *PPP* purchasing power parity, *GLP1a* Glucagon-like peptide 1 agonists, *DPP4i* Dipeptidyl peptidase-4 inhibitors

The robustness of the pooled INB, as well as heterogeneity, can be assessed using various sensitivity and subgroup analyses. Sensitivity analyses omitting the study that used a societal perspective [38] and the study that did not use discounting [40] yielded a pooled INB of US$ 4,032.07 (US$ − 554.48, US$8,618.61) and US$4,068.19 (US$ − 650.66, US$8787.04), respectively.

The WTP threshold used for these comparisons ranged from US$ 29,382 to US$ 58,024, with a median of US $49,325. Subgroup analyses by median WTP threshold (< vs ≥ US $49,325), time horizon, and source of effectiveness measure were performed (see Table 4), indicating GLP1s were not significantly cost-effective compared with DPP4i in any subgroup. In all these sensitivity and subgroup analyses, the results were similar to the overall pooled INB, indicating that the results are robust.

**Table 4:** The subgroup analysis results of pooing INB between the GLP1 agonists and DPP4 inhibitors

Subgroup analysis	No. of comparisons	Pooled INB (US$)	95% CI	p-value	I^2^ (%)
**Threshold**					
< Median ($49,325)	5	3,554.00	-1,825.34 to 8933.34	0.829	0.0
≥ Median ($49,325)	5	5,226.56	-3530.74 to 13,983.86	0.393	2.4
**Time Horizon**					
Life time	7	2,663.36	-2463.30 to 7790.01	0.852	0.0
Non-lifetime	3	9,386.72	-846.73 to 19,620.17	0.424	0.0
**Source of effectiveness**					
Multiple study	4	1,538.51	-8,049.60 to 11,126.62	0.742	0.0
Single study	6	4,745.01	-473.58 to 9,963.59	0.534	0.0

As in general MA, publication bias was assessed using a funnel plot and Egger’s test. There was no evidence of publication bias, seen either by asymmetry on the funnel plot (Fig. 2b) or an Egger’s test (coefficient = 0.32, SE = 0.73, *p* = 0.672).

## Discussion

We have extended the COMER MA methods for EESs, focusing on data harmonisation; methodological issues include currency, time, discount, perspective, time horizon, and model used to aid in applying a MA for evidence synthesis in EESs. INB and its variance are estimated based on five scenarios. MA is then applied to pool INBs across studies providing a summary estimated CE of treatment relative to control. This evidence should be useful for policymakers in making decisions regarding reimbursement of treatments to a population in countries where resources are limited.

Despite the existence of several guidelines for reporting EESs, studies still vary in how they report the results[44]. This data harmonisation process reported here under the five scenarios can help prepare data to calculate and pool INB values. The different monetary units and year can all be converted to a common standard currency.

We used INB instead of ICER as the economic effect measure following COMER methods because of limitations of the ICER in the ambiguity of interpretation for negative ICER as mentioned above [8, 13]. On the other hand, positive INBs indicate cost-effectiveness, while negative INBs show non-cost-effectiveness. This information will be required by policymakers [14, 15] in making a decision from both resource-rich and resource-poor countries.

A few challenges should be highlighted when applying a MA in EESs. First, EEs are heterogeneous, which can be caused by model type, population, country income, GDP, perspective, time horizon, and discount rate. We applied the CPI and PPP to harmonise different economic backgrounds as well as the time-lag across the studies [45, 46]. However, it should be noted that using CPI and/or PPP may have some limitations as for the estimation method of price indices, which are calculated from individual prices of only selected commodities rather than all commodities in each country [47]. Considering not only country income but also model type, time horizon, and perspective in stratified analyses may also reduce heterogeneity, if there are sufficient data for stratifying. Furthermore, sub-group and/or sensitivity analyses should be performed to identify specific types of studies/country income where treatments show cost-effectiveness. Economic factors should be considered for subgroups, including WTP, discount rate, type of EES (e.g., within-trial EES versus model-based EES), quality of EESs or risk of bias, the structure of economic model, type of health state, and percent herd immunity for the vaccine, etc.). Different subgroups of these factors may result in different cost-effectiveness findings within HICs and UMICs.

Second, the health EESs are context-specific, usually conducted in individual country settings. However, not all countries have EESs that fit their context because conducting well-designed EESs is very resource-intensive and requires specialised expertise in economic evaluation. Therefore, there will be an even greater need for some systematic synthesis of evidence where resources are limited. Evidence from a MA of EESs will be useful if it is performed with sensitivity to country contexts (e.g., country income, type of model, lifetime, perspective, etc.).

In conclusion, we have described a tutorial of MA in EESs by applying the general methods of MA, additional with specific issues for EESs. The step-by-step approach of data harmonization is demonstrated for facilitating the process of MA. Although evidence of CE is context-specific for each country, conducting such specific individual study is challenging as similar to CE studies due to various practical limitations (e.g., trained manpower, time, resources, etc.). Thus, the MA of EESs should be encouraged; evidence synthesis would be of immense value for the policy decision-making process as well as aid in the comparability of such evidences across countries with similar contexts.

## Supplementary Information

**Additional file 1**.

## Data Availability

All the relevant data is included in the manuscript.

## Abbreviations

ΔC: Incremental cost
ΔE: Incremental effectiveness
BMI: Body mass index
CE: Cost effectiveness
CE-plane: Cost effectiveness plane
CI: Confidence interval
CPI: Consumer price index
CEA: Cost effectiveness analysis
COMER: COMparative Efficiency Research
CUA: Cost utility study
DALY: Disability adjusted life year
DPP4i: Dipeptidyl peptidase 4 inhibitors
EES: Economic evaluation studies
GDP: Gross domestic product
GLP1i: Glucagon-like peptide-1
HIC: High income countries
ICER: Incremental cost effectiveness ratio
INB: Incremental net benefit
LIC: Low income countries
MA: Meta analysis
PPP: Purchasing power parity
PRISMA-P: Preferred Reporting Items for Systematic Reviews and Meta-Analyses protocol
PSA: Probabilistic sensitivity analysis
SD: Standard deviation
SE: Standard error
QALY: Quality adjusted life years
UMIC: Upper middle-income countries
US: United states
WHO: World health organisation
WTP: Willingness to pay
