# Supplementary material for: Meta-analysis of economic evaluation studies: data harmonisation and methodological issues
Source: BMC Health Serv Res. 2022 Feb 15;22:202. doi: 10.1186/s12913-022-07595-1 (PMC8845252; doi:10.1186/s12913-022-07595-1)
Supplement: Supplementary file 1 — Additional file 1. [file 12913_2022_7595_MOESM1_ESM.docx]

**APPENDIX**

**DATA EXTRACTION FORM**

**Part I General Article Information**

1. Date of data extraction // (DD/MM/YYYY)

2. Study ID 

3. Reviewer  1. ………….. 2. ………….

4. First Author and Email of contact author:_______________________________________

5. Journal __________________________________________________

6. Year of publication 

**Part II General Study characteristics**

7. Country ________________________

8. Setting  1. Country  2. University hospital  3. Tertiary hospital  4. Others________

9. Study perspective  1. Societal  2. Healthcare payer’s  3. Others ______________

10. Type of EEs  1. CUA  2. CEA  3. CBA

11. Study design  1. Alongside Cohort  2. Alongside RCT  3. Model based

1. 12. Funding 1. Yes ______________ 2. No 3. Not mentioned
2. 13. Conflicts of interest 1. Yes _______________ 2. No 3. Not mentioned

**Part III Characteristics of studied participants, intervention**

14. Target population  1. T2DM with dual therapy  2. T2DM triple therapy

15. Sample size (n)  (for alongside trial)

16. Mean age (years) .

17. Gender male % .

18. Intervention(s)/comparator(s)

 1. Dapagliflozin  2a. Cana_100  2b. Cana_300

3. Empagliflozin  4. SGLT2i  5.Others____________________

**Part IV Methods and outcomes of economic evaluations**

19. Time horizon  1. Lifetime  2. Others specified  years

20. Type of model  1. Markov  2. Decision tree  3. Discrete event simulation

 4. Not specified  5. None (alongside trial)  6. Others_____________

21. Name of Model:______________________

22. Discount rate  1. Yes  2. No

23. Discount rate for costs . %

24. Discount rate for effects . %

25. Reference (Currency) year of analysis 

26. Currency ________________________________

27. Category of costs  1. DMC  2. DNMC  3. IDC  4. Not given

28. Data source of cost  1. Elicited in the study  2. Administrative data

 3. Clinical database  4. Medical record

 5. Published literature  6. Others ____________________

29. Data source of utility  1. Elicited in the study  2. Systematic review

 3. Other study/literature  4. Not done

30. Data source of effectiveness

 1. Single study based clinical effectiveness

 2. Multiple study based clinical effectiveness

 3. Synthesis based estimates

31. Analysis of uncertainty  1. One-way sensitivity analysis  2. Probabilistic

 3. Others ________________  4. Not done

32. Threshold Used for ICER:  1. Country specific  2. GDP based

33. Threshold in currency: _________________

34. Literacy rate of the country:

35. Type of outcome measures reported

[ ] Cost [ ] LY [ ] QALY [ ] ICER

**Part V Findings and data for pooling**

1. **Findings of study**

| Scenario | Intervention: | Comparators: | Result /Conclusion (Domi./Cost E/Not CE) |
| --- | --- | --- | --- |
| I |  |  |  |
| II |  |  |  |
| III |  |  |  |
| IV |  |  |  |

1. Data used for pooling

| **I. Base-case (Deterministic)** | **Intervention** | **Comparator** | **Comments** |  |
| --- | --- | --- | --- | --- |
| Costs |  |  |  |  |
| LY |  |  |  |  |
| QALY |  |  |  |  |
| Incremental LY |  | |  |  |
| Incremental QALY |  | |  |  |
| ICER |  | |  |  |
| Other |  |  |  |  |
| Other |  |  |  |  |
| Other |  |  |  |  |
| PSA details: (No of iterations/populations): | | | | |
| **II. PSA-based** | **Intervention**  **Mean ± SD or**  **95% CI** | **Comparator**  **Mean ± SD or**  **95% CI** | **Comments** |  |
| Costs |  |  |  |  |
| LY |  |  |  |  |
| QALY |  |  |  |  |
| Incremental LY |  | |  |  |
| Incremental QALY |  | |  |  |
| ICER |  | |  |  |
| Other |  |  |  |  |
| Other |  |  |  |  |

**Comments/Notes:__________________________________________________________**

Supplement Table 1: The costs and QALYs as reported in the primary studies in Scenario -3

| **Author** | Intervention Cost | Comparator Cost | Intervention QALY | Comparator QALY | Incremental cost | Incremental QALY | ICER per QALY |
| --- | --- | --- | --- | --- | --- | --- | --- |
| Davies MJ (2012) | **21793±544** | **19951±521** | **7.52±0.11** | **7.34±0.11** | **1842±751** | **0.19±0.15** | **10158** |
| Lee WC (2012) | **81444±1079** | **76262±1061** | **8.825±0.117** | **8.624±0.115** | **5182** | **0.201** | **31488** |
| Mezquita Raya P (2013) | **54684±1250** | **52387±1346** | **9.04±0.13** | **8.87±0.11** | **2297** | **0.17** | **13266** |
| Perez A (2015) | **56628±1323** | **52450±1394** | **9.239±0.121** | **8.838±0.121** | **4177** | **0.4** | **10436** |
| Bruhn D (2016) | **140806±1948** | **138583±2071** | **9.618±0.125** | **9.517±0.130** | **2223** | **0.101** | **22094** |
| Roussel R (2016) | **43031±1532** | **40472±1513** | **10.09±0.13** | **9.84±0.13** | **2558(2427 to 2689)** | **0.25 (0.24 to 0.26)** | **10275** |
| Barnett AH (2018) | **24737±739** | **22362±725** | **9.18±0.12** | **9.02±0.11** | **2375** | **0.15** | **15423** |
